# The Landscape of Novel Therapeutics and Challenges in Glioblastoma Multiforme: Contemporary State and Future Directions

**DOI:** 10.3390/ph13110389

**Published:** 2020-11-14

**Authors:** Karam Khaddour, Tanner M. Johanns, George Ansstas

**Affiliations:** 1 Division of Hematology and Oncology, University of Illinois at Chicago, Chicago, IL 60612, USA; 2 Division of Medical Oncology, Washington University School of Medicine, Saint Louis, MO 63110, USA; tannerjohanns@wustl.edu

**Keywords:** glioblastoma multiforme, immunotherapy, immune checkpoint inhibitors, vaccine, CART therapy, oncolytic virus, immunosuppressive

## Abstract

Background: Glioblastoma multiforme is a malignant intracranial neoplasm that constitutes a therapeutic challenge because of the associated high morbidity and mortality given the lack of effective approved medication and aggressive nature of the tumor. However, there has been extensive research recently to address the reasons implicated in the resistant nature of the tumor to pharmaceutical compounds, which have resulted in several clinical trials investigating promising treatment approaches. Methods: We reviewed literature published since 2010 from PUBMED and several annual meeting abstracts through 15 September 2020. Selected articles included those relevant to topics of glioblastoma tumor biology, original basic research, clinical trials, seminal reviews, and meta-analyses. We provide a discussion based on the collected evidence regarding the challenging factors encountered during treatment, and we highlighted the relevant trials of novel therapies including immunotherapy and targeted medication. Results: Selected literature revealed four main factors implicated in the low efficacy encountered with investigational treatments which included: (1) blood-brain barrier; (2) immunosuppressive microenvironment; (3) genetic heterogeneity; (4) external factors related to previous systemic treatment that can modulate tumor microenvironment. Investigational therapies discussed in this review were classified as immunotherapy and targeted therapy. Immunotherapy included: (1) immune checkpoint inhibitors; (2) adoptive cell transfer therapy; (3) therapeutic vaccines; (4) oncolytic virus therapy. Targeted therapy included tyrosine kinase inhibitors and other receptor inhibitors. Finally, we provide our perspective on future directions in treatment of glioblastoma. Conclusion: Despite the limited success in development of effective therapeutics in glioblastoma, many treatment approaches hold potential promise including immunotherapy and novel combinational drugs. Addressing the molecular landscape and resistant immunosuppressive nature of glioblastoma are imperative in further development of effective treatments.

## 1. Introduction

**Figure 1:**
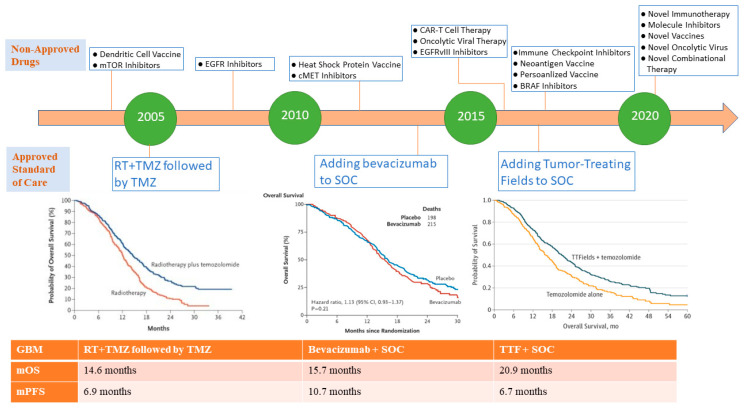
Timeline of treatment approval for glioblastoma and landmark investigational non-approved drugs. The lower panel demonstrates Kaplan–Meier curves of three pivotal randomized controlled trials in newly diagnosed glioblastoma which led to the approval of standard of care systemic treatment including concurrent radiation and temozolomide (Stupp protocol) [7], addition of bevacizumab to Stupp protocol [10,11], and use of tumor-treating fields with Stupp protocol [8]. Despite the improvement of OS with the addition of temzolomide, bevacizumab and TTF, the overall survival remains poor (as noted by the continued drop in slope of the curves). There are no approved treatments shown to improve the survival in recurrent glioblastoma (not shown in this figure) [12]. Kaplan–Meier curves reprinted by permission from the Massachusetts Medical Society (NEJM) and American Medical Association (JAMA). N Engl J Med. Radiotherapy plus concomitant and adjuvant temozolomide for glioblastoma. Stupp R, Mason WP, van den Bent MJ, Weller M, Fisher B, Taphoorn MJ, Belanger K et al., copyright © 2005. N Engl J Med. A randomized trial of bevacizumab for newly diagnosed glioblastoma. Gilbert MR, Dignam JJ, Armstrong TS, Wefel JS, Blumenthal DT, Vogelbaum MA et al., copyright © 2014. JAMA. Effect of Tumor-Treating Fields Plus Maintenance Temozolomide vs. Maintenance Temozolomide Alone on Survival in Patients With Glioblastoma: A Randomized Clinical Trial. Stupp R, Taillibert S, Kanner A, Read W, Steinberg D, Lhermitte B et al., copyright © 2017. Abbreviations: TMZ: temozolomide, mOS: median overall survival, mPFS: median progression free survival, RT: radiation therapy, SOC: standard of care, TTF: tumor-treating fields.

Glioblastoma multiforme is the most aggressive intracranial tumor; histologically considered as a malignant glioma arising from an astrocytic lineage (WHO classification: astrocytoma grade IV), and it represents the most common malignant primary brain tumor in adults [1,2]. Despite the low incidence rate of glioblastoma compared to other cancer types, it portends an extremely poor prognosis with a median overall survival (OS) of approximately 1 year, and a relative survival rate at 5 and 10 years approximately (5% and 3%, respectively) [3,4]. Glioblastoma is considered a privileged tumor because of its isolation from the surrounding structures, and the presence of the blood brain barrier (BBB) which impedes the passage of several immune cells and chemotherapeutics [5]. In addition, the heterogeneous genetic and molecular landscape of glioblastoma constitutes a challenge in the face of developing effective therapies, which is reflected by the lack of drug approval in the past decade [6]. The cornerstone of glioblastoma management relies on maximal surgical resection of the tumor with concurrent chemoradiation using the alkylating agent temozolomide (TMZ) followed by adjuvant TMZ for a total of 6 months according to the landmark trial by Stupp et al. [7]. The other treatment modality that has shown improved OS in glioblastoma patients is the addition of tumor treating field (TTF) to the current standard of care (Stupp protocol), which is a device worn by the patient on the scalp, and works by delivering alternating electrical fields that disrupt the microtubules in the mitotic spindle leading to tumor cell death [8]. However, this treatment has proved to be challenging for patients given the low compliance rates with its use and high cost [9]. The addition of bevacizumab which is a vascular endothelial growth factor (VEGF) inhibitor, in newly diagnosed and recurrent glioblastoma-selected patients demonstrated modest improvement in progression-free survival (PFS) and better quality of life, but was associated with a high rate of adverse events without an improvement in OS [10,11,12]. As the majority of glioblastoma ultimately recur during or after treatment, it is imperative to develop therapeutic drugs that could offer clinical benefit to these patients to improve survival, and decrease disease associated morbidity and complications. This unmet need has led to extensive preclinical research to further characterize the molecular and genetic alterations that control tumor growth, as well as the interaction between the immune system and glioblastoma in order to identify candidate targets for effective drug development. This comprehensive effort in the last decade has substantially expanded our understanding of glioblastoma and has been previously reviewed in depth [13,14]. Nevertheless, the limited drug approval for glioblastoma relative to the novel investigational therapies that have been tested, and the modest improvement in OS achieved in the past decade demonstrate the obligation for further research in the field of neuro-oncology to develop new therapies that can improve patient outcomes as outlined in Figure 1. Recently, there have been promising novel drugs including targeted molecular therapy and immunotherapy that have established a possible role in anti-tumor response in glioblastoma. Moreover, enhanced drug delivery across BBB and combination treatment modalities seem to hold a prospective role in the treatment of glioblastoma.

In this review, we discuss the distinct factors that are essential in understanding the challenges in the treatment of glioblastoma. Second, we offer a review of potential novel treatments including (immunotherapy and targeted therapy), and the rationale behind their use. Finally, we present our prospective on future directions in the pursuit of novel effective treatments for glioblastoma.

## 2. Exploiting the Blood-Brain Barrier and the Immune System against Glioblastoma: The Wrong Notion of the Immune Desert in Glioblastoma

Perhaps the most consequential discovery that has shifted the paradigm of cancer treatment and substantially led to improved outcomes in multiple cancer types is the discovery of the immune system role in preventing tumor growth and killing cancer cells which has coined the term “anti-tumor immunity” [15]. For example, checkpoint receptors are membrane-bound proteins that are found on malignant cells as well as immune cells such as T-cell subsets, natural killer (NK) cells, antigen presenting cells (APC) including macrophages and dendritic cells. These checkpoint receptors, are usually upregulated and highly expressed in the tumor microenvironment (TME) of several tumor types, and when bound to their respective ligands are responsible for negative regulation of immune cells leading to increased immune evasion by the tumor through the disruption of innate and adaptive anti-tumor function [16,17,18]. This has resulted in the development of several monoclonal antibodies directed at checkpoint receptors such as cytotoxic-associated lymphocyte antigen-4 (CTLA-4) and programmed death-1/programmed death-ligand 1 (PD-1/PD-L1) [19]. This class of medication known as immune checkpoint inhibitor (ICI) has demonstrated a significant prolonged PFS and OS in several tumors including melanoma, lung, genitourinary, liver, and breast cancers [20,21,22,23,24,25,26,27]. Other checkpoint inhibitors are currently being investigated such as inhibitors of T-cell immunoglobulin and mucin-domain containing-3 (TIM3), lymphocyte activation gene-3 (LAG3), and indoleamine 2,3-dioxygenase-1 (IDO1) [28,29]. Another example that demonstrated the utility of immune derived cells in the treatment of cancer was the use of bioengineered chimeric antigen receptor loaded on autologous T-cells (CAR-T), which have shown efficacy and improved outcomes in relapsed acute lymphoblastic leukemia, refractory diffuse large-B cell lymphoma (DLBCL), and relapsed follicular lymphoma [30,31]. Moreover, the efficacy of oncolytic virus therapy such as talimogene laherparevec (TVEC) in melanoma and autologous dendritic vaccination therapy in prostate cancer have further extended the advent of immunotherapy in the treatment of cancer [32,33]. As such, several immunotherapies that recruit the immune system against cancer cells have been investigated in different tumor types including glioblastoma. To this end, glioblastoma has been regarded for a long time as an isolated tumor encased by BBB, which makes it devoid of active immune cells. This notion has been abandoned in the past decade after several preclinical and clinical studies demonstrated the role of the immune system in the TME of intracranial tumors including glioblastoma. Comprehensive analysis of the role of the immune system in glioblastoma is out of the scope of this review and has been summarized in detail elsewhere [14,34]. To better explain the current challenges in implementing effective immunotherapies for glioblastoma, it is imperative to understand the cross-talk between malignant cells of glioblastoma and immune cells and the immunosuppressive nature of the tumor. Several factors govern this interaction, and can be classified into the following: (1) factors related to BBB; (2) factors related to the immunosuppressive microenvironment; (3) factors related to tumor heterogeneity and genetic signature of glioblastoma subtypes; and (4) extrinsic factors related to the modulating effect of previous systemic treatment.

### 2.1. Factors Related to BBB

The blood-brain barrier (BBB) forms a dynamic interaction of endothelial cells, pericytes, microglia, astrocytes, and is responsible for the integrity of the central nervous system [35]. BBB plays an essential role in regulating the passage of water, ions, immune cells, nutrients, and therapeutic drugs through its complex physical barrier manifested by tight junctions and enzymatic–metabolic pathways that control transportation of different molecules. This can hinder the delivery of therapeutic drugs into the brain compartment as it is estimated that BBB restricts the penetration of 98% of small molecules and 100% of large molecules [36]. In glioblastoma, the integrity of the BBB is disrupted, which leads to alterations involving the vascular bed. The aggressive nature of glioblastoma leads to invasion of endothelial cells and breakage of tight junctions which results in a breach in the BBB [37,38]. Nevertheless, this disruption is not sufficient to allow increased concentration of therapeutic compounds in the tumor bed. Moreover, glioblastoma invasion into the surrounding structures is associated with angiogenesis because of hypoxia-induced VEGF formation, which though could potentially increase drug availability via the tumor vasculature, it also leads to pathological alterations in the BBB and suboptimal drug concentration in tumor foci outside the disrupted region [39]. The constellation of pathophysiological alterations in the vasculature bed associated with glioblastoma has coined the term blood-brain tumor barrier (BBTB) because of its unique features compared to BBB. To this end, analyzing the structure and changes associated with BBTB can aid in effective treatment development for glioblastoma and is an area for active research [40].

Of importance to immunotherapy, BBB plays an essential role in regulating immunosurveillance and restricting immune cell passage into the central nervous system (CNS), which could limit the efficacy of many novel drugs that work by recruiting the immune system to attack cancer cells. However, the insight gained from other CNS diseases on the ability of immune cells to cross BBB in certain autoimmune and inflammatory diseases, could help further research on exploiting this vulnerability in the administration of novel immunotherapies in glioblastoma [41,42,43].

To circumvent these challenges, several methods are under ongoing investigation to overcome the BBB and BBTB and achieve sufficient delivery of therapeutic compounds inside glioblastoma. One promising method is the delivery of drugs through nanoparticles which allow for the individual units loaded with drug to be carried to glioblastoma cells [44]. For example, nano-immunoconjugates (anti CTLA-4 and anti PD-1) on natural biopolymer scaffold were able to induce immune response in glioma cells and lead to a significant prolonged survival in mice with intracranial GL261 glioblastoma [45]. Similarly, another group of investigators showed that the delivery of small interfering RNAs (siRNA) against both epidermal growth factor receptor (EGFR) and PD-L1 through solid lipid nanoparticles in glioblastoma mouse models, was able to decrease the growth of glioblastoma and prolong mouse survival [46].

Convection-enhanced delivery (CED) is another promising method that has shown to encourage preclinical and clinical results [47]. This method relies on establishing a pressure gradient during interstitial infusion of the pharmaceutical compound to augment its distribution into the intracranial tumor to achieve enhanced local drug delivery. One recent example of the clinical benefit associated with CED is the work by Desjardins et al. that showed mild increased OS in patients with recurrent glioblastoma who received intertumoral recombinant nonpathogenic polio-rhinovirus chimera (PVSRIPO) versus matching historical control (median OS 12.5 months vs. 11.3 months, respectively) [48]. Nevertheless, further research is essential to evaluate the safety and efficacy of CED to better identify the best drugs to use with this method as well as unifying treatment protocols and ascertaining the delivery of the infused molecule into tumor bed.

Moreover, it is important to note that some drugs can have an effect on glioblastoma despite not being able to penetrate BBB into the tumor. For example, bevacizumab, which is a VEGF inhibitor that has shown improved PFS and quality of life in glioblastoma patients, prevents angiogenesis within the vasculature compartment without the need of being present inside the tumor cells [10,11,12]. Another example is the use of ICI that can restore anti-tumor efficacy by blocking checkpoint receptors present not only in the tumor milieu, but also in the benign peripheral lymphatic system [49]. This warrants further investigation on novel treatments that could have a potential effect on glioblastoma without the need to penetrate BBB.

### 2.2. Factors Related to Glioblastoma Immunosuppressive Microenvironment

The long-standing historical notion of the CNS being considered an immune desert because it is devoid of effective resident immune cells has been challenged by rigorous research which demonstrated that the CNS has specific lymphatic system and is occupied by immune cells with unique characteristics compared to its counterparts in other organs [50].

The paramount advances in cancer treatment with the introduction of ICI as well as other immunotherapies have drastically shifted the focus in cancer treatment on exploiting pathways involved in host immune defense mechanisms to fight cancer cells “anti-tumor immunity.”

Although immunotherapies have shown significantly improved outcomes in different solid and hematological malignancies, the outcomes in glioblastoma have been mild to moderate at best. This stems from the fact that TME of glioblastoma employs an immunosuppressive effect despite the abundance of a variety of immune cell subsets [14,51]. For example, despite the presence of a functional lymphatic system in the CNS that provides the potential for tumor antigen to prime cognate T cells that could mediate anti-tumor immunity, glioblastoma exerts an inhibitory effect on the functionality of T cells through several mechanisms [52,53]. Moreover, glioblastoma can disrupt the function of non-resident T cells beyond the tumor bed and can affect potential effective peripheral T-cell populations and lead to their entrapment in the bone marrow because of the loss of spingosine-1-phosphate receptor 1 (S1P1) [54]. By contrast, glioblastoma allows the expansion of specific T-cell subsets such as T-regulatory (Treg) cells which are responsible for immune tolerance and promotion of tumor growth [55]. In addition, natural killer (NK) cells which are innate-like immune cells equipped with potent cytotoxic activity against tumor cells are suppressed in glioblastoma [56].

Additionally, tumor-associated macrophages (TAM) and microglia are essential subtypes of immune cells that constitute the majority of cellular mass in glioblastoma (30–50%) [57]. These cells are considered antigen presenting cells (APC) and are responsible for the presentation of tumor-associated antigens to be recognized by effector T cells [57]. TAMs constitute the majority of APC in glioblastoma while microglia account for an additional 15% of APCs [58,59]. Both TAMs and microglia are implicated in gliomagenesis in glioblastoma through several mechanisms including immune suppression and secretion of several soluble factors such as transforming growth factor-β (TGF-β) and interleukin-10 (IL-10) that can further exacerbate immunosuppressive state [60,61,62]. Furthermore, antigen presentation processes are impaired in glioblastoma because of impaired upregulation of major histocompatibility complex (MHC) class II [63].

The other important factor that is associated with an immunosuppressive microenvironment in glioblastoma is the increased expression of checkpoint receptors such as PD-L1, IDO, TIM3 on T-cells and TAMs [64,65,66,67,68,69,70]. Similarly, glioblastoma favors the expression of several inhibitory molecules such as signal transducer and activator of transcription 3 (STAT3) and Fas ligand (FasL) which aggravate immune inhibitory signals and promote T-cell apoptosis, respectively [71,72].

For the abovementioned reasons, the negative impact of glioblastoma on the immune microenvironment which favors the presence of dysfunctional and deregulated immune cell subpopulations, constitute a major challenge on the implementation of effective immunotherapies in the treatment of glioblastoma. Therefore, efforts are focused on exploiting existing vulnerabilities in the TME of glioblastoma to reactivate the immune system against tumor cells. For example, TAMs display plasticity which refers to their ability of switching their phenotypic features leading to a change in their function. As such, this plasticity could be exploited to selectively express a phenotype that favors macrophage type-1 (M1) which is a pro-inflammatory cell that can boost host immunity versus macrophage type-2 (M2) which is considered pro-tumorigenic [73]. Similarly, the increased expression of checkpoint receptors in TME of glioblastoma can be targeted with the use of combinational ICI to rescue effector T-cells and to restore immune surveillance in glioblastoma.

### 2.3. Factors Related to Tumor Heterogeneity and Genetic Signature of Glioblastoma Subtypes

The common definition of glioblastoma by World Health Organization classification as a high-grade glioma-IV offers limited insight into the genetic heterogeneity of glioblastoma. Genomic characterization by The Cancer Genome Atlas (TCGA) research group and the comprehensive molecular analysis helped delineate three distinct tumor-intrinsic transcriptional glioblastoma subtypes including proneural, mesenchymal, and classical [74,75]. This classification relies on the predominant genetic signature associated with each subtype. For example, while the proneural subtype of glioblastoma is associated with amplification in platelet-derived growth factor receptor alpha (PDGFRA), the mesenchymal subtype can be associated with alteration in neurofibromatosis type 1 (NF1), and classical subtype is associated with amplification in EGFR [76]. This is of interest given the evidence that specific expression patterns due to amplification in PDFGRA and EGFR could serve as prognostic biomarkers [77,78]. In addition, some specific immune cell types such as TAM have been shown to have higher expression of their related genes in the mesenchymal subtype of glioblastoma [79].

The genetic heterogeneity appears to reflect distinct glioblastoma immune subsets based on the molecular signature [80]. For example, glioblastoma with wild-type isocitrate dehydrogenase (IDH) status is associated with a higher tumor infiltrative lymphocyte (TIL) and higher PD-L1 expression compared to IDH mutated glioblastoma [81]. Similarly, there is an increased PD-L1 expression on both resident T cells and circulating monocytes in glioblastoma with PTEN loss [82]. In addition, the glioblastoma mesenchymal subtype that is associated with NF-1 and RB-1 mutations can have significantly increased TIL [83]. This suggests that specific genetic alterations can be associated with an abundance of immune cells and increased expression of checkpoint receptors which could help select subgroups of glioblastoma patients who could derive a better response to immunotherapy. However, clinical correlation is still lacking with some evidence showing no predictive value of increased TIL or checkpoint receptors in glioblastoma subsets with improved response to ICI [84].

Furthermore, tumor mutation burden (TMB) status that estimates the frequency of mutations that are present per megabase of malignant cell DNA correlates with response observed in several solid malignancies during treatment with ICI. This is the result of increased neoantigen formation and presence of new mutant proteins that are immunogenic and can induce anti-tumor response [85]. As such, glioblastoma patients with high TMB could reflect a subpopulation that can have better response to immunotherapies such as ICI compared to glioblastoma patients with low TMB. This is of importance given that glioblastoma patients have low TMB at baseline which can limit anti-tumor immunity and explain weak response to immunotherapy [86]. Interestingly, there is a subset of patients with glioblastoma that can have hypermutated status with high TMB after treatment with TMZ and another subset of patients who have germline mutations leading to hypermutated status [87,88,89,90,91,92].

Therefore, further characterization of immune abundance based on gene expression of glioblastoma can prove to be helpful in identifying selected patients as immunotherapy continues to assume a critical role in the treatment of cancer. The correlation between glioblastoma genetic subtypes and tumor response to immunotherapy is an open area for research as deeper understanding is required to evaluate the effect of molecular variations on tumor cell survival and sensitivity or resistance to immunotherapy.

### 2.4. Extrinsic Factors Related to the Microenvironment Modulating Effect of Previous Glioblastoma Treatments

Several iatrogenic factors can affect and lead to alteration of glioblastoma on the genomic level as well as TME. As mentioned in the previous section, treatment with TMZ can increase hypermutated status, which is associated with higher neoantigen formation [87,88,89,90]. In addition, TMZ which is known to cause lymphopenia during treatment, has been shown to have an immunomodulatory effect which could enhance anti-tumor immune response [93]. In contrast, steroids which are commonly used in glioblastoma patients to treat tumor associated vasogenic edema and mass effect can lead to lymphodepletion and favor a more immunosuppressive environment which could hinder efficacy of novel drug effects such as immunotherapy [94,95]. Moreover, the use of glucocorticoids before or during treatment with ICI in different solid tumors is associated with a decreased response and worse OS [96]. This is of importance, as most patients with glioblastoma are treated with dexamethasone and are expected to have a degree of persistent immunosuppression that could counteract the efficacy of immunotherapies.

Additionally, bevacizumab which is used in the treatment of primary and recurrent glioblastoma can lead to diminished infiltrative immune suppressive cells, low PD-1/PD-L1 expression and increased cytotoxic T cells which can alter the efficacy of immunotherapies [97]. To this end, highlighting the influence of previous medications on TME of glioblastoma and subsequent treatments such as immunotherapy, is imperative when evaluating the efficacy of novel glioblastoma therapeutics [98].

## 3. Immunotherapy in the Treatment of Glioblastoma

Immunotherapy entails several pharmaceutical compounds that have different mechanism of action and can be categorized into: (1) Monoclonal antibodies (ICI) that target checkpoint protein receptors (CTLA-4, PD-1, PD-L1, TIM3, IDO, LAG3); (2) adoptive transfer of bioengineered immune cells which are designed to destroy malignant cells such as CAR-T cell therapy and CAR-natural killer therapy (CAR-NK); (3) cancer vaccines that use mutant tumor peptides or immune cell-based components to induce anti-tumor immunity; and (4) oncolytic viruses that preferentially destroy malignant cells through direct and indirect mechanisms by engaging effector immune cells against cancer cells.

### 3.1. Immune Checkpoint Inhibitors in Glioblastoma

Immune checkpoint inhibitors (ICIs) have revolutionized the management of cancer in the last decade since the approval of CTLA-4 inhibitor (ipilimumab) in 2011, which was followed by approval of other ICI (PD-1/PD-L1 inhibitors). As mentioned previously, ICI can reinvigorate anti-tumor immune response by blocking checkpoint receptor molecules which are responsible for an immune-inhibitory effect that leads to immune evasion of cancer cells. The clinical efficacy of this class of medication has been echoed by durable improved outcomes including prolonged OS and PFS in several solid tumors including melanoma, lung, urothelial, breast, and liver cancers. Following the successful implementation of ICI in several solid malignancies, further research was conducted to evaluate their efficacy in other tumor types including glioblastoma. As the main target of ICI is the checkpoint receptor in TME, the presence of these receptors appears to be essential to enhance the ability of these drugs to interrupt checkpoint protein interaction with the respective ligand receptor which eventually lead to reactivation of anti-tumor response [99]. This was reflected by several clinical trials which showed that the expression of a checkpoint receptor (PD-L1) correlated with improved outcomes in patients with metastatic lung and urothelial cancer who received PD-1/PD-L1 inhibitors (though this correlation can vary depending on the choice of ICI and tumor type) [100]. As such, a recent study demonstrated an increased expression of the checkpoint receptor PD-L1 in 90% of 135 patients who were diagnosed with glioblastoma [66]. This serves as a proof of concept of a potential role of ICI in treatment of glioblastoma because of the presence of targetable checkpoint receptors in TME. Consequentially, the presence of checkpoint receptors in glioblastoma appears to amplify immunosuppression and correlate with worse prognosis. For example, a study by Nduom et al. showed that expression of PD-L1 in 94 glioblastoma patients correlated with increased risk of death (hazards ratio [HR] = 1.54, *p* = 0.0343) [101]. Similarly, high expression of other checkpoint receptors such as CTLA-4, TIM3, and IDO was found to correlate with negative outcomes and decreased survival [70,102,103].

The enthusiasm to carry out the outstanding results achieved in cancer management with ICI, has not been as successful as anticipated in glioblastoma trials. This was demonstrated in a pivotal phase III randomized controlled trial (CheckMate-143), which compared the safety and efficacy of nivolumab (PD-1 inhibitor) versus bevacizumab in patients with recurrent glioblastoma who were previously treated with TMZ and radiotherapy at initial diagnosis [104]. This study demonstrated a lower objective response rate, lower PFS, similar median OS, and higher treatment-related adverse events in patients who received nivolumab compared to those who received bevacizumab [104]. Another phase-3 trial evaluating the addition of nivolumab to concurrent chemoradiation (TMZ and radiotherapy) in newly diagnosed MGMT-unmethylated glioblastoma failed to demonstrate improved outcomes [105]. Similarly, another PD-1 inhibitor (pembrolizumab) did not achieve improved PFS at 6 months or improved OS in a phase II study evaluating pembrolizumab versus pembrolizumab and bevacizumab in recurrent glioblastoma [106]. Despite the disappointing results of these clinical trials, they provided important clues which further helped in designing new trials testing different approaches of ICI treatment in glioblastoma.

One of the factors that is proposed to contribute to the decreased response of ICI is its inability to reach tumor cells. This is of importance as the BBB allows passage of limited small size compounds less than 400 Da, whereas nivolumab and pembrolizumab have a molecular weight of 146 kDA and 149 kDa respectively [107,108,109]. As such, there are several groups working on enhancing the delivery of these pharmaceuticals through the BBB into the TME. Some of this work focuses on nanoparticle delivery systems as described before [45], and other groups are investigating combining ICI with radiotherapy to increase the disruption of BBB (ClinicalTrials.gov Identifier: NCT02311582).

Another factor that appears to be fundamental for the efficacy of ICI in treatment of glioblastoma is the timing of administration. Most of the trials to date have examined efficacy of ICI either in the recurrent setting or in first line setting in combination with concurrent chemoradiation after surgical resection of the tumor. Therefore, it was tempting to evaluate the use of ICI prior to surgery as they have demonstrated improved outcomes in the neo-adjuvant setting in different tumors such as breast cancer and melanoma [110]. Several advantages can be achieved with neo-adjuvant administration of ICI including higher T-cell expansion in these patients compared to those who receive ICI after surgical resection of the tumor [111]. This is hypothesized to be the result of a large tumor bulk presence that contains potential high load of immunogenic antigens, which can elicit a more robust anti-tumor response in the presence of ICI. As such, the efficacy of ICI in the post-surgical setting could attenuate the anti-tumor immune response because of the unavailability of tumor antigens after surgical resection of the tumor. The advantage of neo-adjuvant ICI has been validated in recurrent glioblastoma in several studies. Cloughesy et al. randomized 35 patients with recurrent glioblastoma to receive either neo-adjuvant pembrolizumab (one single dose intravenously 2 weeks prior to surgery) and/or adjuvant ICI [112]. The study highlighted the role of neo-adjuvant ICI in upregulating T-cell populations and interferon-γ-related gene expression compared to subjects who did not receive neo-adjuvant pembrolizumab [112]. This also correlated with an observed median OS of approximately 14 months in patients who received neo-adjuvant pembrolizumab versus 7.5 months in those who did not receive ICI prior to surgical resection [112]. Similarly, another phase-II trial replicated similar findings in patients with recurrent glioblastoma who received neo-adjuvant nivolumab prior to surgical resection [113]. The administration of nivolumab before surgery was associated with a higher immune cell activity in the TME, higher tumor-infiltrating lymphocytes, and enhanced expression of chemokine transcripts, however, this trial did not result in prolonged OS [113]. To this end, the sequence of ICI administration in regards to surgery in glioblastoma appears to be imperative for the enhancement of the functional immune landscape leading to a substantial tumor response and seem to be promising, which led to several clinical trials that are ongoing to address this approach.

Furthermore, the potential role of ICI in the treatment of glioblastoma appears to be significant in specific subpopulations. For example, tumor mutation burden (TMB) and mismatch repair deficiency (MRD) reflect a state in which a tumor is loaded with a high number of mutated proteins and neoantigens given the defective DNA repair mechanisms. This can convert the tumor into a hotspot as a target for anti-tumor immunity mediated by ICI as higher number of immunogenic antigens are expected to elicit a strong immune response [114,115]. Despite the low frequency of both TMB and MRD in glioblastoma, some patients have been described to have defective DNA repair mechanisms leading to an immune permissive TME, which provides an advantage for ICI in priming an anti-glioblastoma immune response [116,117]. To illustrate this, Bouffet et al. reported a durable response with the use of nivolumab in two pediatric siblings with recurrent glioblastoma who had biallelic MRD [92]. Similarly, another report demonstrated a durable response to pembrolizumab in an adult patient with recurrent glioblastoma who had germline polymerase DNA epsilon (POLE) deficiency which correlated with a high tumor mutation load [91]. To this end, it is important to highlight that hypermutation status and high tumor mutation burden do not confer a better response when they develop after the administration of TMZ. This has been demonstrated in a study by Touat et al. that analyzed hypermutation and TMB status in 10,294 gliomas and found that hypermutation status post-TMZ was associated with lower abundance of tumor infiltrative lymphocytes and worse outcomes [118]. This highlights the difference between the hypermutation induced by germline deficiency in DNA repair mechanisms versus hypermutation induced by post-TMZ use. In addition, specific mutations in glioblastoma have not been reported to be associated with improved response when treated with ICI as demonstrated by Zhao et al. who reported increased and prolonged response to PD-1 inhibitors in glioblastoma patients who had B-raf murine sarcoma (BRAF) and protein tyrosine phosphatase non-receptor type 11 (PTPN11)-activating mutations [84]. This collective evidence points toward more individualized trial design and further investigation into genomic characteristics to identify potential patients who could be candidates for ICI therapy.

**Figure 2:**
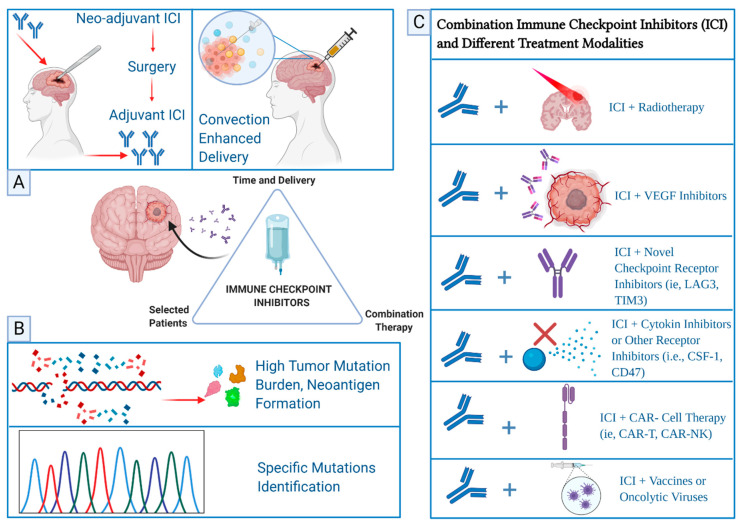
Proposed approaches for the improvement of glioblastoma treatment with immune checkpoint inhibitors (ICI) include timing of administration and method of delivery, patient selection, and combinatorial therapies. Panel (**A**) demonstrates the promising approach of administering ICI in the neo-adjuvant setting prior to surgical resection of glioblastoma and followed by adjuvant ICI [112,113]. Convection enhanced delivery of nanoparticle ICI is another method that could improve the availability and action of ICI in glioblastoma [44]. Panel (**B**) shows that proper patient selection for treatment with ICI could be of a clinical benefit such as patients with high tumor mutation burden due to germline deficiency affecting DNA repair mechanisms and in patients with specific mutations that could attenuate the immunosuppressive effect in the microenvironment of the tumor [84,91,92]. Panel (**C**) illustrates ongoing investigational combination of ICI with other novel therapies.

Current efforts are focused on combining ICI with different treatment modalities in glioblastoma such as the novel checkpoint inhibitors of LAG3 and TIM3 [119,120]. Interestingly, a variety of molecules and receptors have emerged recently as potential targets in combination with ICI such as TGF-β inhibitors, colony-stimulating factor-1 ligand (CSF-1) inhibitors, and CD47 blockade [121,122]. Other interesting approaches studying the effect of epigenetic in glioblastoma have been suggested such as the use of micro RNA (miRNA) modulation effect on the TME as a biomarker to predict response and resistance to ICI to guide treatment and has been reviewed [123]. The ongoing research on identifying selected patient population, identifying the right sequence of treatment, and combining ICI with other investigational therapeutics seem to hold a high promise in further advancement of glioblastoma care Figure 2.

### 3.2. Adoptive Cell Therapy in Glioblastoma

Adoptive cell therapy in cancer treatment relies on redirecting immune cells to engage with specific tumor antigens based on bioengineered chimeric antigen receptors expressed in autologous T cells [124]. By targeting tumor antigens, bioengineered T-cells are able to initiate an augmented anti-tumor response through the activation of a variety of effector immune cells. This approach has shown promising results in hematological malignancies, which has generated enthusiasm toward applying this new approach in glioblastoma especially given that there was a signal of response observed in patients with secondary CNS lymphoma who received CAR-T therapy [125]. This therapy was initially investigated in glioblastoma to evaluate safety due to the known severe adverse event of immune effector-associated neurological syndrome (ICANS) which can increase toxicity and morbidities in glioblastoma patients who already have neurologic deficits. To this end, Brown et al. reported the safety of administering CAR IL13Rα2 expressed on cytotoxic T-lymphocyte clones in patients with recurrent glioblastoma [126]. The latter work has led to a report of a significant clinical response in a patient with recurrent glioblastoma who was treated with IL13BBζ–CAR T cells with sustained disease control that lasted 7.5 months but later progressed to four different new locations [127]. Other investigators explored targeting different glioblastoma-related tumor cell receptors such as epidermal growth factor receptor variant-III (EGFRvIII) and human epidermal receptor-2 (HER2) as they are commonly expressed on glioblastoma cells. These studies demonstrated safety of EGFRvIII-CAR and HER2- CAR-T infusions and an active anti-tumor effect, however, this was yet to demonstrate a clinical benefit with an improved survival [128,129,130]. Another approach of adoptive cell therapy that has just entered early clinical investigation involves the administration of bioengineered NK cells [131]. The advantage of using NK cells compared to CAR-T cell therapy relies on the fact that NK cells are leveraged to retain high cytotoxic effect against tumor cells irrespective of specific antigen expression which could lead to an increased efficacy against antigenically heterogeneous tumor cells [131]. Nevertheless, the immature results of CAR-cell-based therapy in glioblastoma require further evidence and investigation to support safety and efficacy in glioblastoma. Among the important factors to address during the ongoing research on CAR-cell therapy in glioblastoma include: finding a common targetable antigen that is universally expressed on tumor cells and is involved in driving tumorigenesis, expanding the repertoire of the bioengineered cells to target more than one antigen such as using bi-specific CAR-T-cell therapy and acceptable safety [132].

### 3.3. Therapeutic Vaccine Therapy in Glioblastoma

Therapeutic cancer vaccination relies on the method of inducing an anti-tumor immune response by selecting tumor-associated antigens or mutated peptides that are specific for cancer cells and are not universally expressed in healthy tissue. These candidate antigens are then either enhanced ex vivo or incubated in specific immune cells like dendritic cells and then reintroduced into the patient. By doing so, immunogenic tumor-associated antigens are hypothesized to initiate a tumor-specific immune response that can contribute to regression of malignant cells [133]. The platform of therapeutic vaccines in glioblastoma utilizes different approaches and can be classified into: (a) Tumor antigen associated vaccines that use commonly expressed oncogene-derived proteins on tumor cell surface such as the use of EGFRvIII and IDH1(R132H) to elicit a specific immune response; (b) vaccines that use heat shock protein family to illicit an immune response against a variety of different tumor antigens; (c) cell-based vaccine using autologous or allogenic dendritic cell (DC) which serves in initiating anti-tumor response through enhanced antigen presentation and; (d) personalized vaccines using immunogenomic methods to detect patient-specific novel peptides which are considered to be immunogenic neoantigen to enhance the response against malignant cells.

Vaccine-based therapies using EGFRvIII have generated a lot of enthusiasm as extracellular mutations of EGFRvIII are commonly expressed in glioblastoma (approximately 20%) and are responsible for constitutive EGFR pathway activation [134]. Early phase-II clinical trial conducted by Schuster et al. demonstrated safety and increased immunogenicity of EGFRvIII peptide sequence conjugated to keyhole limpet hemocyanin when administered with Stupp protocol in newly diagnosed glioblastoma [135]. However, despite the early encouraging results, a phase-III randomized controlled trial failed to demonstrate survival advantage of the candidate vaccine [136]. Interestingly, a signal of clinical benefit was observed in the previous trial when a post-hoc analysis showed improved OS in patients with bulky glioblastoma who received the vaccines although this was not the primary end point of the study [136]. In addition, a recent randomized phase-II trial demonstrated improved PFS in patients with EGFRvIII-positive recurrent glioblastoma when they received EGFRvIII vaccine in combination with bevacizumab versus those receiving placebo-bevacizumab [137]. To this end, EGFRvIII vaccines could assume a future role in the treatment of glioblastoma based on an enhanced clinical signal observed in specific subpopulations or when the vaccine is used in combination with other treatment modalities, although further confirmatory large randomized trials will be needed. Another, potential mutated protein that is commonly encountered in glioblastoma and has been shown to induce immunogenicity is IDH1 R132H [138]. Early results demonstrated safety and immunogenicity of a vaccine targeting this mutation in a German phase I clinical trial in 32 patients with newly diagnosed IDH1 R132H mutant malignant astrocytoma [139].

While vaccines against EGFRvIII and IDH1 R132H target specifically malignant cells that express the mutant protein, vaccination using heat shock protein family can leverage a broader response targeting a variety of tumor antigens in glioblastoma which could mitigate the heterogeneous nature of the tumor as a resistance mechanism. This is hypothesized to be the result of the function of heat shock protein family in post translational protein processes and presentation of antigens on MHC class I [140]. Several groups have demonstrated relative safety and immunogenicity using heat shock protein family vaccines in newly diagnosed and recurrent glioblastoma but data is still immature to support their clinical utility [141,142,143].

The rationale of using DC vaccines stems from their unique characteristics as highly potent cells that have the capacity to present antigens. The presentation of antigens by DC leads to sensitization of naïve T cells and priming of an anti-tumor immune response. The potential benefit of dendritic cell vaccination in newly diagnosed and recurrent glioblastoma has been illustrated in a systematic review that included a total of 403 patients and showed improved OS in patients with glioblastoma who received DC vaccines compared to historical average survival [144]. A randomized phase-III trial by Liau et al. have further corroborated the evidence of a benefit with the addition of autologous tumor lysate-pulsed DC vaccine (DCVax-L) to Stupp protocol in patients with newly diagnosed glioblastoma which has led to the approval of this vaccine in Switzerland [145].

Finally, personalized vaccines using newly detected neoantigens is another promising avenue in glioblastoma research. This personalized vaccine approach is based on the fact that neoantigens are composed of immunogenic-mutated peptides that are the product of genomic disruption in glioblastoma cells and when expressed in the TME can lead to an anti-tumor response [146]. For example, the use of personalized vaccine composed of DCVax followed by a neoantigen-based synthetic long peptide vaccine demonstrated immunogenicity of such an approach in a patient with newly diagnosed glioblastoma [147]. Of interest, two recent clinical trials have further demonstrated the immunogenic potential and clinical efficacy using whole-exome sequencing or microarray analysis in identifying candidate neoantigen peptide vaccines that were shown to generate anti-tumor response [148,149].

Key challenges remain when developing therapeutic glioblastoma vaccines including heterogeneity of glioblastoma as well as the immunosuppressive environment, which constitute a major hurdle given their direct dependency on antigen-specific derived immune response. The ongoing work evaluating variable vaccines in glioblastoma treatment should offer further insight.

### 3.4. Oncolytic Virus Therapy

Oncolytic viral therapy in glioblastoma involves the intratumoral administration of genetically modified viruses that usually have neural tropism and an ability to selectively replicate inside malignant infected cells. The amplified replication of lytic viruses leads to the destruction of the target cell and further propagation of viral progeny in addition to intracellular molecules, which can induce and augment anti-tumor immune response [150,151]. Some tumor-selective lytic viruses have shown ability to replicate in glioma cells and are currently being studied in early phase clinical trials including herpes simplex virus (HSV), adenovirus, poliovirus, gamma-retrovirus, and zika virus [150,152,153]. The method of administration is by intraparenchymal or intracranial arterial convection-enhanced delivery which allows for the direct intratumoral administration of the oncolytic virus. Although the majority of the results of this therapeutic avenue remains confined to early phase clinical trials, the results to date have demonstrated accepted safety and efficacy of this approach. For example, Ji et al. reported the results of administration of intra-arterial cerebral infusion of adenovirus mutant thymidine kinase in 53 patients with recurrent glioblastoma and showed improved PFS and OS compared to control group [154]. This study demonstrated acceptable safety compared to control groups with the exception of one patient who developed vasospasm 10 days after oncolytic viral administration [154]. Similarly, Cloughesy et al. demonstrated tolerability of using retroviral replicating virus (Toca511) followed by administration of Toca FC which led to the preferential killing of infected cancer cells through delivery of a yeast cytosine deaminase, which in turn converts 5-fluorocytosine to 5-fluorouracil (a chemotherapeutic compound that inhibits tumor cell growth and leads to cytotoxicity) [155]. In this phase I, open-label trial, 45 patients with recurrent glioblastoma received the viral therapy. The trials demonstrated the safety of this approach and an OS survival benefit although it was not the primary endpoint of this study [155]. As mentioned previously, Desjardins et al. demonstrated safety and significant OS improvement in patients with recurrent glioblastoma who received a recombinant oncolytic polio-rhinovirus (PVSRIPO) with an OS rate of 21% at 24 months which was maintained at 36 months [48]. The promising efficacy of oncolytic viral therapy in glioblastoma treatment led to FDA breakthrough designation for PVSRIPO in 2016. Recently, the overall survival rate at 36 months from Desjardins group (OS rate of 20%) was replicated by Lang et al. who administered an oncolytic-replicating adenovirus (DNX-2401) in 25 patients with recurrent glioblastoma [156]. Interestingly, it was observed that there was a decrease in the exhaustion of TIM3 and increased infiltration by CD8+ T cells of tumor specimens after the treatment with the oncolytic vaccine which has led to another clinical trial evaluating the combination of this vaccine with PD-1 inhibitor (NCT02798406). This cumulative evidence opens a new avenue for the immunotherapies in glioblastoma although it is essential to confirm the previous findings in large phase 3 randomized trials.

## 4. Targeted Therapies in Glioblastoma

**Figure 3:**
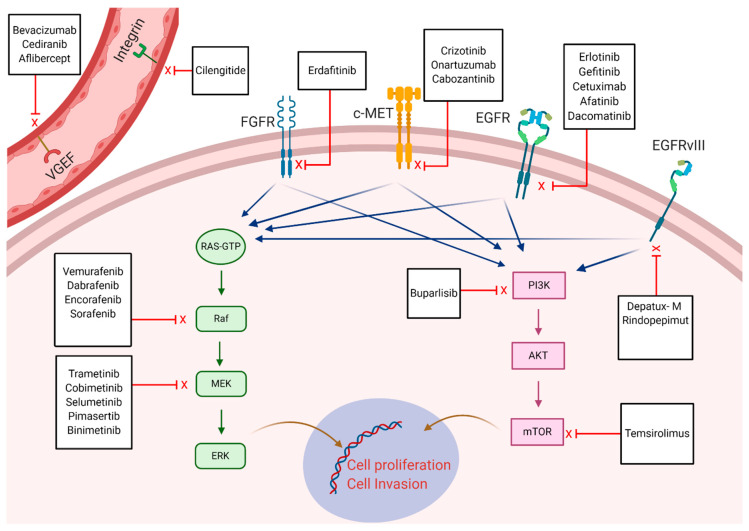
Targeting pathways and receptors involved in cell proliferation, aggressiveness, invasion, and angiogenesis in glioblastoma. Some of the pharmaceuticals which have been trialed to target these specific receptors or pathways are illustrated.

The advent of molecular targeted therapy has revamped cancer treatment since their introduction in early 2000. Their efficacy relies on targeting specific molecular alterations or their protein products which govern malignant cell growth and enhance the invasive nature of the tumor. Most of the targeted therapies are tyrosine kinase inhibitors (TKIs) that can prevent kinase phosphorylation which are implicated in cancer cell proliferation and angiogenesis [157]. Conversely, drugs that can block receptors in the TME involved in tumor growth such as VEGF inhibitors are considered targeted therapies. The successful use of these targeted therapies in multiple solid and hematological malignancies has persuaded the investigation of their efficacy in glioblastoma. To this point, glioblastoma can be associated with a variety of mutations which has been described by The Cancer Genome Atlas research network [134]. This has resulted in several investigations which have been conducted to evaluate the outcomes in glioblastoma patients treated with specific molecular targets. Variable drugs have been investigated and were directed against mutations involving EGFR, fibroblast growth factor receptor (FGFR), MET, BRAF-MAPK, PI3K-AKT mTOR pathway, VEGF, and integrins Figure 3.

Frequency of EGFR amplification is estimated to reach 40%, and in-frame deletions (EGFRvIII) in approximately 25% [134]. The alterations in EGFR can lead to constitutive activation of cell proliferation and contribute to prognosis which makes it a plausible target in specific tumors such as breast and lung cancer. However, it appears that EGFR-associated alterations in glioblastoma lack prognostic significance, which is multifactorial because of the difference in structural mutation location (extracellular in glioblastoma versus intracellular in lung cancer) as well as the presence of regulatory downstream circuits that can lead to cell growth independent of EGFR status [158,159]. As such, majority of clinical trials failed to demonstrate improved PFS or OS in recurrent patients treated with different EGFR inhibitors including erlotinib, gefitinib, afatinib, and dacomitinib [160,161,162,163]. The failure of the previous therapeutics has shifted the focus toward different strategies using EGFR receptors as binding sites on the tumor cells for the delivery of cytotoxic payload. Lassman et al. demonstrated safety in 60 patients with recurrent glioblastoma who received the antibody–drug conjugate depatuxizumab mafodotin (depatux-m) [164]. However, a more recent phase-II trial using depatux-m in combination with TMZ at progression did not improve OS significantly compared to the control arm [165].

The presence of FGFR gene fusion was investigated as a target for therapy despite its low frequency (3.5%) in glioblastoma which is exclusive to IDH1/2 wild-type tumors [166]. In the previous study, two patients with FGFR fusion gene were treated with erdafitinib (FGFR inhibitor), and showed promising tumor response but both eventually had progressive disease [166]. Similarly, targeting c-MET overexpression (frequency of 13% in glioblastoma) and amplification (5%) with two phase II trials in recurrent glioblastoma, failed to demonstrate improved outcomes [167,168,169].

Targeting the PI3K-AKT-mTOR pathway which is universally altered in glioblastoma has also been unsuccessful and showed no improved outcomes in both newly diagnosed and recurrent glioblastoma using PI3K inhibitors and mTOR inhibitors [170,171,172]. Interestingly, despite the low frequency of BRAF^V600E^ mutation in glioblastoma (up to 3%), some subtypes of glioblastoma such as epithelioid glioblastoma show high frequency reaching 50% [173,174]. Based on the efficacy of targeting mutations in the MAPK pathway including BRAF^V600E^ in melanoma, several anecdotal reports described rapid and durable anti-tumor response using inhibitors of this pathways such as BRAF and MEK inhibitors [175,176]. A study by Kaley et al. has further corroborated the efficacy of BRAF inhibitors in glioblastoma patients [177]. Nevertheless, utility and access to these medications remain scarce given that most of the patients eventually develop progressive disease while on treatment and because of the restricted number of eligible patients with such mutation.

Finally, other study groups attempted targeting receptors in the TME of glioblastoma which are not necessarily expressed on tumor cells such as VEGF and integrins. The trials using the anti-VEGF (bevacizumab) later led to its approval in the treatment of glioblastoma as described earlier in the introduction. Several trials evaluated the efficacy of several integrin inhibitors but failed to show any benefit of the addition of these targeted therapies and have been summarized in detail by Le Rhun et al. [178].

Collectively, the failure of most targeted therapy trials to date in treatment of glioblastoma has resulted in the decline in the interest of targeted molecular therapy with the exception of novel molecular targets that are under active research [178]. Supplementary research remains essential to identify pathways and molecular alterations that control tumor survival and resistant nature of glioblastoma in order to develop further effective therapeutics.

## 5. Future Perspective of Glioblastoma Research

Despite the increased armamentarium of effective cancer therapeutics across multiple tumor types, management of glioblastoma continues to prove the unmet need for further research to develop new effective medication, which is echoed by the high number of clinical trials that are under way (470 clinical trials registered at clinicaltrials.gov as of October 2020). Phase 3 randomized clinical trials are crucial for evaluating the role of novel therapies in glioblastoma despite the promising results of early phase clinical trials.

Addressing the immunological landscape and the crosstalk between intracranial glioblastoma and the extracranial immune components appear to be of a high priority given that most of the concentration revolves around exploiting the immune system against glioblastoma. Achieving clarity on the utility of immunotherapy (ICI) up-front in the neo-adjuvant setting is promising but requires further validation in large prospective randomized trials. In addition, several proposed approaches have been suggested to overcome the immunosuppressive TME in glioblastoma and are currently under investigation (summarized in Table 1). 

Furthermore, combinatorial therapies with immunotherapies is currently being explored and appear to be promising. For example, inclusion of poly-ADP ribose polymerase (PARP) inhibitors which induces synthetic lethality and leads to overload with defective DNA repair mechanisms appears to be promising. Preclinical studies suggest that PARP inhibition with drugs such as olaparib can increase neoantigen formation, lead to PD-L1 upregulation, boost type I Interferon signaling, and reprogram TME to facilitate anti-tumor response when using ICI [179]. In addition, PARP inhibition is thought to cause catastrophic DNA damage which may also lead to resensitized tumor cells to TMZ in MRD glioblastoma [180]. Similarly, Hanna et al. demonstrated in a phase I trial the radio-sensitizing effect and the safety of olaparib administration with TMZ prior to surgery in patients with recurrent glioblastoma [181].

Ultimately, as immunotherapy continues to dominate the research arena in glioblastoma therapeutic development, it would be important to stratify patients based on their previous glucocorticoid use as most of the patients require to be on steroids which is implicated in suppression of the immune system and can counteract the effect of drugs targeting the immune system.

## 6. Conclusions

The lack of drug approvals for the treatment of glioblastoma and the mild to modest improvement in survival in the past decade represent a growing need for well-designed research to develop effective therapeutics. The ongoing effort should consider the factors that are implicated in the resistance observed with these novel investigational therapeutics to further leverage our understanding and optimize our treatment approach. Immunotherapy holds a promise in glioblastoma despite the immunosuppressive nature of the tumor. Combination immunotherapy with other treatment modalities, investigational novel compounds, and innovative delivery modalities such as antibody–drug conjugates, convection-enhanced delivery, and nanoparticles are currently under ongoing research which will address their safety and efficacy in glioblastoma. Further characterization of patients’ tumor molecular profiles can help identify subpopulations who could derive a benefit from currently available treatments.

## Figures and Tables

**Table 1:** Factors implicated in the immunosuppressive environment of glioblastoma and possible solutions to enhance response.

Factors Related to Glioblastoma	Possible Solutions	Reference
Restricted drug delivery due to blood brain barrier	Convection enhanced delivery (CED) and nano particles. Disruption of blood brain barrier with radiation therapy combined with immune checkpoint inhibitors Adoptive cell therapy	[44,45] [121] [127,128]
Effector T-cell suppression and peripheral T-cell entrapment	Use of immune checkpoint inhibitors, therapeutic vaccines or adoptive cell transfer therapy	[52,53,54,55]
Increased expression of checkpoint receptors	Use of combination checkpoint inhibitors targeting several checkpoint receptors	[64,65,66,67,68,69,70,121]
Suppression of natural killer cells	Use of checkpoint inhibitors Natural killer cell adoptive transfer	[131]
Abundance of tumor associated macrophages (TAM) (protumorigenic phenotype)	Boosting host immunity through exploiting plasticity in TAM to express inflammatory phenotype M1	[73]
Increased secretion of TGF-β	Use of checkpoint inhibitors with TGF-β inhibitors	[121]
